# Heat-Killed *Lactobacillus acidophilus* Promotes Growth by Modulating the Gut Microbiota Composition and Fecal Metabolites of Piglets

**DOI:** 10.3390/ani14172528

**Published:** 2024-08-30

**Authors:** Huabiao Miao, Jing Liang, Ganqiu Lan, Qian Wu, Zunxi Huang

**Affiliations:** 1 School of Life Science, Yunnan Normal University, Kunming 650500, China; ynsfmhb@user.ynnu.edu.cn (H.M.); wuqian@ynnu.edu.cn (Q.W.); 2 Engineering Research Center for Efficient Utilization of Characteristic Biological Resources in Yunnan, Ministry of Education, Kunming 650500, China; 3 Key Laboratory of Yunnan for Biomass Energy and Biotechnology of Environment, Kunming 650500, China; 4 Laboratory of Animal Genetics and Breeding, College of Animal Science and Technology, Guangxi University, Nanning 530004, China; 18387115331@163.com (J.L.); dsf421503251@163.com (G.L.)

**Keywords:** paraprobiotics, antibiotics, pig nutrition, gut microbiome, metabolite

## Abstract

**Simple Summary:**

This study was the first attempt to use heat-killed *Lactobacillus acidophilus* as a feed additive to replace antibiotics. The results of the evaluation showed that it could improve the performance of piglets, increase intestinal microbial diversity and thus ensure the intestinal health of piglets. In conclusion, this provides a reference for the application of heat-killed *Lactobacillus acidophilus*.

**Abstract:**

Probiotics can improve animal growth performance and intestinal health. However, understanding the effects of paraprobiotics on the growth performance and gut microbiota of piglets and how the paraprobiotics exert their impact are still limited. The present study was conducted to investigate the effects of heat-killed *Lactobacillus acidophilus* IFFI 6005 supplementation on the growth performance, intestinal microbiota, and fecal metabolites of piglets. First, a feed-additive sample of heat-killed *Lactobacillus acidophilus* IFFI 6005 was prepared by culture. Second, 96 (initial BW = 14.38 ± 0.67 kg, weaning age of 40 days) healthy piglets were selected and randomized into four treatment groups. Each treatment group consisted of three replicates (*n* = 8). Pigs were fed a basal diet (NC), basal diet plus antibiotics (PC), basal diet plus *Lactobacillus acidophilus* IFFI 6005 at 600 g/t (LA, 1.0 × 10^10^ cfu/g), and basal diet plus heat-killed *Lactobacillus acidophilus* IFFI 6005 at 600 g/t (HKLA), respectively; the trial lasted for 30 days. The results showed that the ratios of feed to gain (F:G) and diarrhea rate of both the HKLA and PC groups were significantly lower compared with the NC and LA groups (*p* < 0.05); however, there was no significant difference between the HKLA and PC group (*p* > 0.05). In addition, the average daily weight gain (ADG) of the HKLA group was significantly higher (*p* < 0.05) than that of the other three groups in terms of growth performance. Finally, 16S rRNA sequencing and metabolome analysis based on fecal samples further elaborated that the addition of heat-killed *Lactobacillus acidophilus* IFFI 6005 to the feed improved the intestinal microbial diversity and abundance (*p* < 0.05) and reduced the abundance of pathogenic bacteria (*p* < 0.05), but it did not affect the abundance of *Lactobacillus* (*p* > 0.05). Through the comparison of microbial abundance and metabolite content between the two groups (NC_vs_HKLA), the largest differences were found in six microorganisms and 10 metabolites in the intestine (*p* < 0.05). These differential metabolites were involved in the digestion, absorption and utilization of protein and starch, as well as in oxidative stress. In summary, addition of heat-killed *Lactobacillus acidophilus* IFFI 6005 as a new feed additive in piglets has beneficial effects on the growth performance, intestinal bacteria and metabolites, and can be used as an alternative to antibiotics.

## 1. Introduction

Antibiotics are commonly employed in traditional pig farming to prevent and treat diseases. Nevertheless, the improper use of antibiotics can contribute to the emergence of pathogen resistance, diminishing the effectiveness of treatment [1]. Furthermore, the misuse of antibiotics disturbs the intestinal microbiota and modifies the microbiological system, ultimately affecting the growth, digestion, and immunity of weaned piglets [2]. Thus, it is necessary to explore alternative approaches that minimize the reliance on antibiotics in pig farming.

The concept of probiotics, defined as “live microorganisms that offer health benefits to the host when administered in adequate quantities”, is widely recognized [3]. Previous studies have shown that incorporating probiotics into pig diets can enhance immune responses, improve digestion through enzyme activities, and promote growth performance [4]. Nevertheless, the use of live probiotics remains controversial due to their interaction with the ecosystem [5]. Moreover, ensuring the viability and metabolic and functional activities of probiotics throughout their shelf-life is crucial, especially for lactic acid bacteria, and the high-temperature environment, preparation methods, feed storage, and ability to survive passage through the gastrointestinal tract are major challenges [6]. Microencapsulated probiotics can reduce the negative impact of the environment on probiotics, improve the stability of probiotics, and reduce the damage and death of probiotics during processing and storage and in the gastrointestinal environment, but there is no doubt that this will increase costs [7]. Recent research indicated that paraprobiotics can yield similar advantageous outcomes as live probiotics [8]. In addition to regulating immunity and deterring pathogens by increasing intestinal adhesion, paraprobiotics offer the host benefits through metabolites released from deceased cells. Notably, they facilitate easier storage, longer product shelf-life, and more convenient transportation compared to live probiotics [9]. Furthermore, paraprobiotics complement the risk factors and stability associated with live probiotics [10].

Heat treatment methods are commonly employed to obtain paraprobiotics, often referred to as heat-killed probiotics. These heat-killed probiotics can release components like lipoteichoic acids, peptidoglycan, or exopolysaccharides, which possess immunoregulatory effects against pathogens [8]. These include but are not limited to heat-killed *Lactiplantibacillus plantarum* L-137, *Lacticaseibacillus rhamnosus* (LR), and *Ligilactobacillus salivarius* strain 189 (HK LS 189), which have been used in animal husbandry and have shown good application results [11,12,13]. There is no doubt that the high-density culture of *Lactobacilli* is the key to the preparation of paraprobiotics, and only when the bacteria grow to a sufficient biomass can more metabolites be released [14]. The composition of the culture medium and the culture conditions are some of the most important conditions for the high-density fermentation of Lactobacilli strains [15].

Therefore, this study aimed to cultivate and prepare heat-killed *Lactobacillus acidophilus* IFFI 6005, which was derived from dairy production, to compare the effects of live and paraprobiotics *Lactobacillus acidophilus* IFFI 6005 on the growth of piglets and to explore the effect of paraprobiotics *Lactobacillus acidophilus* IFFI 6005 on changes in intestinal fecal microbiomes and metabolites profile in piglets. The results of this study may provide essential information for the application of heat-killed *Lactobacillus acidophilus* IFFI 6005 in the development of sustainable pig farming.

## 2. Materials and Methods

### 2.1. Animal Ethics

The experimental procedures conducted in this study were thoroughly reviewed and approved by the Institutional Animal Care and Use Committee of Guangxi University (GXU-2021–053) in compliance with the Regulations for the Administration of Affairs Concerning Experimental Animals (The State Science and Technology Commission of P. R. China 1988).

### 2.2. Preparation of Heat-Killed Lactobacillus acidophilus IFFI 6005

*Lactobacillus acidophilus* IFFI 6005 was used to perform a 50 L scale-up fermentation with a FUS-50 L fermenter (Guoqiang, Shanghai, China). The seeds were inoculated into a fermenter containing 35 L of fresh superrich medium at a 6% inoculated amount. The culture temperature was 40 °C, and the pH was maintained at pH 5.5 by feeding NaOH. The fermenter pressure was maintained at 0.05 MPa, with no aeration, at minimum speed. When the cell growth rate reached the stable phase, the fermentation broth pH was not regulated, and 3 L of feed medium (5% yeast extract and glucose) was fed to the fermenter at a constant flow rate. After 48 h of incubation, the fermenter temperature was raised to 100 °C, and heat-killing was carried out for 2 h at a speed of 300 rpm/min. The heat-killed *Lactobacillus acidophilus* IFFI 6005 fermentation broth was concentrated 10-fold at 50 °C using a single-acting concentrator (Sinobest, Jining, China). The concentrate was then tested for antibacterial activity. The agar well diffusion method was used with blank culture medium and lysed concentrate against Gram-positive bacteria (*Staphylococcus aureus* and *Clostridium perfringens*) and Gram-negative bacteria (*Salmonella enterica* and *Escherichia coli*) [16]. All bacterial strains were procured from the collection center. The concentrate was added to 30% bran powder and corn starch for boiling granulation to obtain a sample of heat-killed *Lactobacillus acidophilus* IFFI 6005 for feed additive.

### 2.3. Animal and Experimental Design

In this study, 96 weaned piglets (initial body weight = 14.38 ± 0.67 kg, weaning age of 40 days, Duroc × [Landrace × Yorkshire]) in good health were randomly assigned to four treatment groups based on similar body weights. Each treatment group consisted of three replicates, and each replicate contained eight weaned piglets housed together in one pen. The dietary treatments comprised of the following: a positive control group (PC) receiving a basal diet supplemented with chlortetracycline at 380 ppm (a prophylactic and growth-promoting dose; Shandong Shengli Bioengineering Co., Ltd., Shandong, Jining, China); a negative control group (NC) receiving a basal diet (no antibiotics and probiotics); a group supplemented with live *Lactobacillus acidophilus* IFFI 6005 (LA), receiving a basal diet enriched with 600 g/T *Lactobacillus acidophilus* IFFI 6005 (1.0 × 10^10^ cfu/g; Kunming Qactive Biotechnology Co., Ltd., Kunming, China), in which the content was determined by the dilution coating plate method [17]; and a test group (HKLA) fed a basal diet + 600 g/T heat-killed *Lactobacillus acidophilus* IFFI 6005, the same strain as the LA group. Table S1 shows the composition and nutrients of the basal diet performed by National Research Council nutrient requirements [18].

### 2.4. Animal Administrations

The 30-day trial was conducted at Songwan Agricultural Development Company’s pig farm in Nanning, Guangxi, from 30 November 2021 to 6 January 2022 (including a seven-day pretest period). The breeding environment was controlled by an automatic environmental control system (Big Dutchman, Vechta, Germany), including a temperature of 24 °C, controlled humidity levels, sufficient oxygen supply for the piglets, and effective removal of carbon dioxide and ammonia throughout the experiment. They were provided with pelleted feed for ad libitum consumption. Daily production management was routinely performed.

### 2.5. Test Indicator Determination

On the first and thirtieth days of the experiment, individual piglets were weighed, and the total feed consumption for each pen was recorded. The average daily weight gain (ADG) = (test final weight–test initial weight)/number of test days; the average daily feed intake (ADFI) = (feed intake during test–feed remaining during test)/number of test days; the ratio of feed to gain (F:G) = average daily feed intake/average daily gain. Diarrhea among piglets was recorded twice a day (morning and afternoon) at regular intervals, and the diarrhea rate was calculated for each treatment group as follows: normal: cylindrically shaped (0 points); soft feces: thin, soft, shapely (1 point); mild diarrhea: sticky, unshapely, porridge-like thin stool (2 points); severe diarrhea: liquid, unshapely, watery thin stool (3 points). Among these, 2 points or more was recorded as diarrhea, and the treatment was recorded in detail. Diarrhea rate = number of pigs with diarrhea/(number of test pigs × total days) × 100 [19].

### 2.6. Fecal Sample Collection and Index Determination

#### 2.6.1. Fecal Sample Collection

On the 30th day of the experiment, the freshly excreted fecal samples were collected from the middle non-contaminated site with a sterile medicine spoon, immediately placed into a 50 mL sterile centrifuge tube, and placed in liquid nitrogen tanks for freezing [20]. Considering the non-universality of the PC group, as there are efforts in gradually replacing antibiotics globally and the growth performance of the LA group did not achieve the desired performance, no fecal samples were collected from those groups. Therefore, six replicates (two replicate samples were taken from each of the three pens in each group) of feces samples were taken from NC (control group) and HKLA (test group), respectively. A total of 12 samples were collected.

#### 2.6.2. Illumina Sequencing

Fecal microbial DNA extraction, amplification, and purification were performed in accordance with the standard steps of Supplementary Materials and methods M1 [18], and sequenced using an Illumina MiSeq PE300 platform (Genedenovo, Guangzhou, China). The sequences and metadata obtained were uploaded to the Omicsmart Cloud Platform (https://www.omicsmart.com/16S/16s_home.html#/OtuFilter, accessed on 27 August 2024). Taxonomic analysis was conducted using the RDP classifier Bayesian algorithm for operational taxonomic units (OTUs) at a 97% similarity threshold [21]. MOTHUR software 1.30.1 (Genedenovo, Guangzhou, China) was employed at each taxonomic level to assess alpha diversity and analyze community composition within each sample group, utilizing the Silva database for reference [22]. Principal Coordinates Analysis (PCoA) plots and Venn diagrams were generated using the R package in the platform [20].

#### 2.6.3. Determination and Analysis of the Metabolome

Fecal samples were treated and then injected into the LC-MS/MS system for analysis. Fecal samples weighing 100 mg each were ground with liquid nitrogen, and the resulting homogenate was resuspended in 500 μL of prechilled 80% methanol by thorough vortexing. After incubating the samples on ice for 5 min, they were centrifuged at 15,000× *g* and 4 °C for 20 min. A portion of the supernatant was diluted with LC-MS-grade water to achieve a final concentration of 53% methanol. The diluted samples were transferred to new Eppendorf tubes and subjected to another round of centrifugation at 15,000× *g* and 4 °C for 20 min. Finally, the resulting supernatant was injected into the LC-MS/MS system for analysis. The UHPLC-MS/MS analyses were performed using a Vanquish UHPLC system (Thermo Fisher, Bremen, Germany) coupled with an Orbitrap Q ExactiveTM HF-X mass spectrometer (Thermo Fisher, Bremen, Germany) at Gene Denovo Co., Ltd. (Guangzhou, China). Samples were introduced onto a Hypesil Gold column (100 × 2.1 mm, 1.9 μm) and subjected to a 17 min linear gradient at a flow rate of 0.2 mL/min. For the positive polarity mode, eluent A (0.1% formic acid in water) and eluent B (methanol) were used as mobile phases. In the negative polarity mode, eluent A (5 mM ammonium acetate, pH 9.0) and eluent B (methanol) were employed. The solvent gradient was programmed as follows: 2% B for 1.5 min, 2–100% B over 12.0 min, 100% B for 14.0 min, 100–2% B at 14.1 min, and 2% B until 17 min. Operating in either positive or negative polarity mode, the Q ExactiveTM HF-X mass spectrometer had a spray voltage of 3.2 kV, a capillary temperature of 320 °C, a sheath gas flow rate of 40 units, and an auxiliary gas flow rate of 10 units.

The raw data files obtained from UHPLC-MS/MS analysis were processed using Compound Discoverer 3.1 (Thermo Fisher, Bremen, Germany) to perform essential tasks such as peak alignment, peak picking, and quantitation for each metabolite [23]. The key parameters were configured as follows: retention time tolerance of 0.2 min, actual mass tolerance of 5 ppm, signal intensity tolerance of 30%, signal-to-noise ratio of 3, and minimum intensity of 100,000. Subsequently, the peak intensities were normalized relative to the total spectral intensity. The normalized data were then utilized for molecular formula prediction based on additive ions, molecular ion peaks, and fragment ions. Furthermore, the peaks were matched against the mzCloud (https://www.mzcloud.org/, accessed on 27 August 2024), mzVault, and Mass List database resources, enabling accurate qualitative and relative quantitative outcomes [23,24]. Statistical analyses were carried out employing popular software tools such as R, Python, and CentOS from the platform [24,25,26,27,28]. In cases where the data did not exhibit a normal distribution, attempts were made to achieve normalization via the area normalization method [23,24].

### 2.7. Integration Analysis between Microbiota and Metabolome

The metabolite abundance dataset and genus-level microbiota dataset were utilized to construct bidirectional orthogonal projections to latent structure (O2PLS) models. The O2PLS models were calculated using the OmicsPLS package in R (Genedenovo, Guangzhou, China) [25]. To assess the relationship between different levels of microbiota and metabolomic datasets, the Pearson correlation coefficient was computed using the psych package in the R project. Furthermore, a correlation heatmap was generated using the pheatmap package in R (Genedenovo, Guangzhou, China), providing a visual representation of the correlations [29].

### 2.8. Statistical Analysis

Preliminary processing of the data was conducted using Excel 2010 (Microsoft, Redmond, WA, USA). One-way analysis of variance (ANOVA) was performed between groups using SPSS 17.0. For multiple comparison tests between groups, Duncan’s method (Version 17.0, SPSS Inc., Chicago, IL, USA) was employed. The results are presented as the mean values accompanied by standard error. To assess the differences between the two groups, a t-test with false discovery rate correction was utilized. A significance level of *p* < 0.05 was considered indicative of a statistically significant difference. The statistical analysis and visualization results of the bioinformatics involved in this study can be reproduced through the Omicsmart Cloud platform.

## 3. Results

### 3.1. Preparation of Heat-Killed Lactobacillus acidophilus IFFI 6005

**Figure 1:**
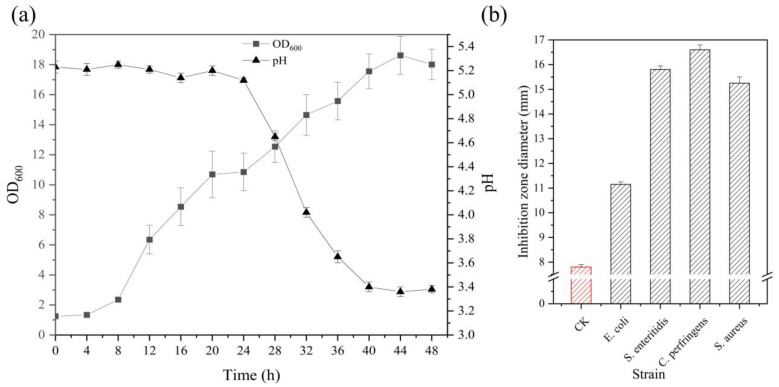
Culture and preparation of heat-killed *Lactobacillus acidophilus*. (**a**) High-density fermentation results of *Lactobacillus acidophilus* IFFI 6005 in a 50-L fermenter. To determine the biomass and pH, culture samples were collected every 4 h from 0 to 48 h. Biomass and pH are represented by squares and triangles, respectively. (**b**) Antibacterial activity of the fermentation concentrate with heat-killed *Lactobacillus acidophilus* IFFI 6005. The test method for antibacterial activity was the agar well diffusion method. CK represents the diameter of the inhibition zone measured by adding an equal amount of blank medium, indicated by a red histogram. The results represent the mean ± SD.

To obtain heat-killed *Lactobacillus acidophilus* IFFI 6005 samples, we performed fermentation using a fed-batch strategy. The maximum OD_600_ in the fermenter was 18.62 at 44 h, and the pH value dropped below 3.5 within 40 h (Figure 1a). After heat killing and concentration, the inhibition zone of the intestinal bacteria *E. coli*, *S. enterica*, *S. aureus,* and *C. perfringens* reached 11.15 mm, 15.80 mm, 16.60 mm, and 18.25 mm, respectively (Figure 1b). These results show that heat-killed *Lactobacillus acidophilus* IFFI 6005 had good antibacterial activity against both Gram-positive and Gram-negative bacteria.

### 3.2. Growth Performance and Diarrhea Rate

During the whole test (from 1 to 30 days), in terms of BW30, the HKLA group was the highest, and the NC group was the lowest compared to the NC group; the BW30 of the HKLA and PC group increased by 8.36% (*p* = 0.0312) and 6.52% (*p* = 0.0449), respectively; and there was no significant difference in the LA group (*p* = 0.7021). In terms of ADG, compared with the NC group, the three test groups increased by 8.01% (*p* = 0.0398), 0.70% (*p* = 0.5982), and 14.07% (*p* = 0.0245), respectively, and HKLA also significantly increased by 5.61% (*p* = 0.0436) compared with the PC group. For ADFI, the HKLA group was the highest, with an increase of 4.78% (*p* = 0.0403) compared to the NC group. For F:G, NC was the highest, and the PC group was the lowest. Compared with the NC group, the PC group and HKLA were decreased by 10.53% (*p* = 0.0147) and 7.89% (*p* = 0.0197), respectively, although the F:G of the HKLA group was higher than that of the PC group, but the difference was not significant (*p* = 0.6924). For diarrhea rate, the PC group and the HKLA group showed a significant decrease, and the HKLA group was the lowest, with a significant decrease of 54.72% (*p* = 0.0249) compared with the NC group (Table 1).

### 3.3. Analysis of the Intestinal Microbiota

#### 3.3.1. Analysis of Alpha Diversity in Intestinal Microorganisms

**Figure 2:**
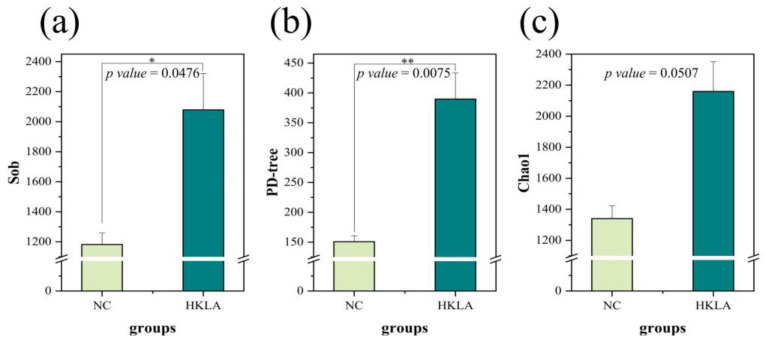
Alpha diversity in intestinal microorganisms from each treatment group (*n* = 6 in each treatment). (**a**) Sob index; (**b**) PD-tree index; (**c**) Chao1 index; abbreviations: NC = control group; HKLA = test group. * and ** represent *p* < 0.05 and *p* < 0.01, respectively, using a *t*-test.

The fecal microbial alpha diversity in each treatment group is shown in Figure 2. Compared with the control group, the Sob (*p* = 0.0476) and PD-tree (*p* = 0.0075) indices of the test group increased significantly (Figure 2a,b). In particular, we found that the Sob index and Chao1 index increased approximately two times in the test group (Figure 2a,c). The results obtained from this study demonstrate that the incorporation of heat-killed *Lactobacillus acidophilus* IFFI 6005 yielded positive effects on enhancing both the diversity and richness of the intestinal microbial community in weaned piglets.

#### 3.3.2. Identification of Target Microorganisms by Differential Analysis

**Figure 3:**
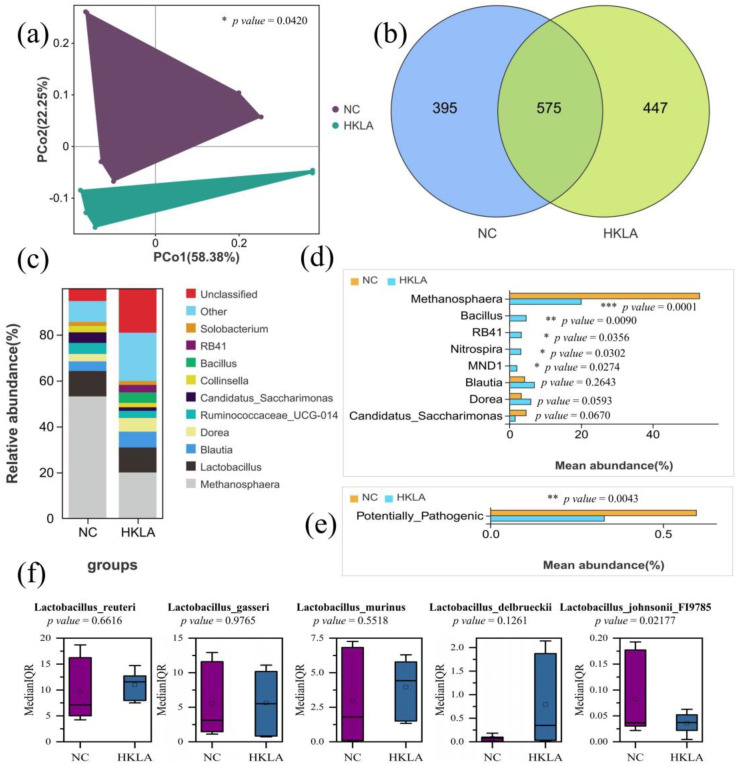
Differential analysis of the microbiota in weaned piglet fecal samples (*n* = 6 in each treatment). (**a**) PCoA of the gut bacterial communities between two groups; (**b**) Venn analysis at the OTU level; (**c**) analysis of species composition of differential operational taxonomic units at the genus level; (**d**) statistical analysis of differential species of abundance; (**e**) the prediction of the function for specific OTUs in the Greengene database. (**f**) Comparison of the contents of the top 5 *Lactobacillus* in the two groups. Abbreviations: NC = control group; HKLA = test group. PCoA = intergroup principal coordinate analysis; PCo1 = the principal coordinate components that explain the largest possible variation in the data; PCo2 = the principal coordinate components that explain the largest proportion of the remaining variability. The mean (SD) values of each group were determined using a Student’s *t*-test. Median (IQR) values were determined using a Mann–Whitney U test. *, **, and *** represent *p* < 0.05, *p* < 0.01, and *p* < 0.001, respectively, using *t*-tests.

The genus-level PCoA showed a significant difference in species similarity between the two groups (*p* = 0.0420) (Figure 3a). Subsequently, the operational taxonomic units of the differences were determined by Venn analysis. The total number of OTUs was 395, which were unique to the NC group, 447 were unique to the test group, and 575 were common to both groups (Figure 3b). Compared to the control group, the unique OTUs and the common OTUs were significantly different between the two groups. Thus, the unique OTUs (Tag ≥ 10) and the common OTUs with significant differences in the two groups were further analyzed.

A total of 89 specific OTUs were discovered, including 38 unique OTUs (Tag ≥ 10; Table S2) and 51 OTUs with significant differences between the two groups (Figure S1). The species composition of these specific OTUs at the genus level was then determined. There were eight genera (*Methanosphaera*, *Blautia*, *Dorea*, *Candidatus_Saccharimonas*, *Bacillus*, *RB41*, *Nitrospira*, and *MND1*) with a relative abundance difference of over 2% between the two groups (Figure 3c). The statistical test results of the relative abundance of microorganisms showed that five differential microorganisms (*Methanosphaera*, *Bacillus*, *RB41*, *Nitrospira*, and *MND1*) were significantly different between the two groups (Figure 3d). Compared with the control group, the relative abundance of *Methanosphaera* (53.12% → 20.02%) in the test group was decreased (*p* = 0.0001), and *Bacillus* (0.06% → 4.62%; *p* = 0.0090), *RB41* (0.02% → 3.26%; *p* = 0.0356), *Nitrospira* (0.02% → 3.13%; *p* = 0.0302), and *MND1* (0.00% → 2.03%; *p* = 0.0274) were significantly increased.

In addition, the prediction of the function for these specific OTUs in the Greengene database showed that the abundance of potentially pathogenic OTUs in the test group decreased significantly compared with that in the control group (*p* = 0.0043; Figure 3e). Interestingly, there was no significant difference in the relative abundance of *Lactobacillus* between the two comparison groups (Figure 3c). In the HKLA group, the relative abundance of the top five *Lactobacillus* (*Limosilactibacillus reuteri*, *Lactobacillus gasseri*, *Ligilactobacillus murinus*, *Lactobacillus delbrueckii*, and *Lactobacillus johnsonii* FI9785) in pigs also showed an upward trend (Figure 3f).

### 3.4. Analysis of Fecal Metabolites

**Figure 4:**
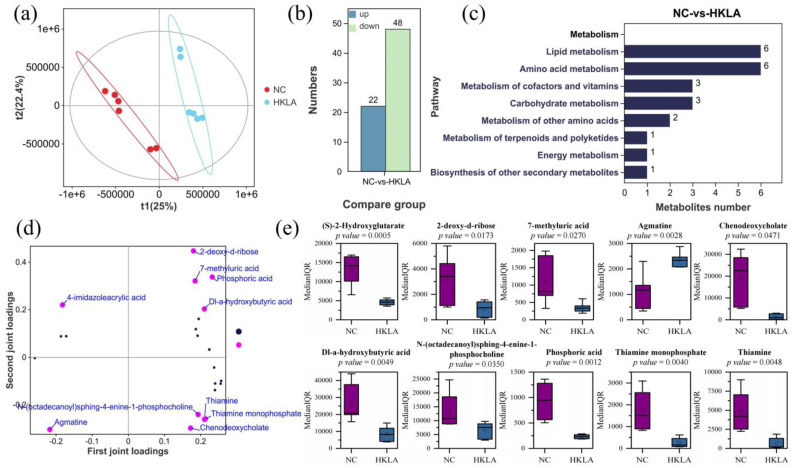
Metabolites in weaned piglet fecal samples (*n* =6 in each treatment). (**a**) Score plot of the PLS-DA model; (**b**) histogram of differential metabolites with *p* < 0.05 and |log_2_(FC)| > 2 between two groups; (**c**) enrichment of differential metabolites in metabolic pathways; (**d**) The key differential metabolite load map; (**e**) top 10 metabolite and bacterial boxplots showed differences between two groups; abbreviations: NC = control group; HKLA = test group. The mean (SD) values of each group were determined using a Student’s *t*-test. Median (IQR) values were determined using a Mann–Whitney U test.

Two groups of fecal samples were analyzed via non-targeted metabolomics, and a total of 2743 endogenous metabolites were obtained. The results of partial least squares-discriminate analysis (PLS-DA) showed that the addition of heat-killed *Lactobacillus acidophilus* IFFI 6005 had a significant effect on the difference of metabolites between the two groups (Figure 4a). Using *p* < 0.05 and |log_2_(FC)| > 2 (FC: fold change) as the screening threshold, 70 metabolites (22 up-regulated and 48 down-regulated) (Table S3) were identified as potential biomarkers (Figure 4b). The functional metabolic pathways of these differential metabolites in the KEGG database were further elucidated. Although these differential metabolites can be enriched into 5 different KEGG A classes (metabolism, human diseases, environmental information processing, organismal systems, and genetic information processing), we only focused on metabolic pathways. A total of 22 differential metabolites were enriched into eight metabolism-related pathways through differential comparison between the two groups (Figure 4c). We constructed an O2PLS model to elucidate the contribution of differential metabolites. The results showed that the top 10 metabolites with the greatest differences between the two groups were 2-deoxy-d-ribose (*p* = 0.0173, loading = 0.58), phosphoric acid (*p* = 0.0012, loading = 0.56), 7-methyluric acid (*p* = 0.0270, loading = 0.51), agmatine (*p* = 0.0028, loading = 0.51), chenodeoxycholate (*p* = 0.0471, loading = 0.50), thiamine monophosphate (*p* = 0.0040, loading = 0.47), thiamine (*p* = 0.0048, loading = 0.46), N-(octadecanoyl)sphing-4-enine-1-phosphocholine (*p* = 0.0350, loading = 0.45), Dl-a-hydroxybutyric acid (*p* = 0.0049, loading = 0.42), and (S)-2-hydroxyglutarate (*p* = 0.0005, loading = 0.37) (Figure 4d,e).

### 3.5. Association Analysis of Differential Microorganisms and Metabolites

**Figure 5:**
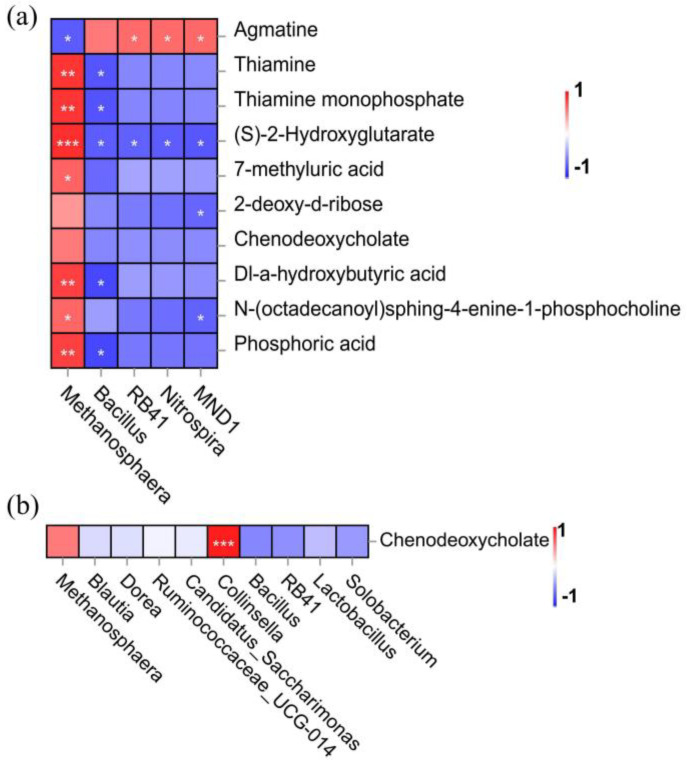
Association analysis of differential microorganisms and metabolites (*n* = 6 in each treatment). (**a**) Correlation analysis of key fecal differential microorganisms with metabolites; (**b**) correlation analysis of the differential metabolite chenodeoxycholate with the top 10 differential microorganisms. The legend shows the magnitude of Spearman’s correlation coefficient; red indicates a positive correlation, and blue indicates a negative correlation. *, **, and *** represent *p* < 0.05, *p* < 0.01, and *p* < 0.001, respectively, using *t*-tests.

To further determine the relationship between differential microorganisms and metabolites, the correlation of differential species and metabolites was analyzed using association heatmaps (Figure 5a). The differential metabolites thiamine, thiamine monophosphate, Dl-a-hydroxybutyric acid, and phosphoric acid showed a significant positive correlation with the abundance change of *Methanosphaera* and a significant negative correlation with Bacillus. 7-Methyluric acid showed a significant positive correlation only with the abundance change of *Methanosphaera* (*p* = 0.0264), and 2-deoxy-d-ribose was negatively correlated only with MND1 (*p* = 0.0481). The differential metabolite agmatine was negatively correlated with *Methanosphaera* (*p* = 0.0291), and *RB41* (*p* = 0.0371), *Nitrospira* (*p* = 0.0360), and *MND1* (*p* = 0.0331) showed a positive correlation. The differential metabolite (S)-2-hydroxyglutarate was positively correlated with *Methanosphaera* (*p* = 0.0008), *Bacillus* (*p* = 0.0306), *RB41* (*p* = 0.0394), and *Nitrospira* (*p* = 0.0305), and *MND1* (*p* = 0.0241) showed a negative correlation. *Methanosphaera* (*p* = 0.0300) and *MND1* (*p* = 0.0403) were jointly associated with the differential metabolite N-(octadecanoyl)sphing-4-enine-1-phosphocholine. The differential metabolite chenodeoxycholate was also not significantly associated with the differential microorganisms (Figure 5a). Further analysis of the association between the differential metabolite chenodeoxycholate and the top 10 differential microorganisms showed that the differential metabolite chenodeoxycholate was significantly positively correlated with *Collinsella* (*p* = 0.0001; Figure 5b). However, *Collinsella* (2.86% → 1.88%) abundance in the test group decreased by 0.98% (*p* = 0.3162) compared to the control group (Figure 3c).

## 4. Discussion

In this study, a heat-killed *Lactobacillus acidophilus* IFFI 6005 with broad-spectrum antimicrobial activity was cultured and prepared, and the addition of heat-killed *Lactobacillus acidophilus* IFFI 6005 was demonstrated to have beneficial effects on the growth performance, intestinal bacteria and metabolites of piglets. Paraprobiotics *Lactobacillus acidophilus* IFFI 6005 can be used as a new feed additive and as an alternative to antibiotics. Thus, several points are worth discussing.

First, *Lactobacillus* encounter three stress factors simultaneously: high concentrations of lactate, high osmotic pressure, and low intracellular pH, especially during exponential growth [30,31]. Additionally, excessively high initial concentrations of nutritional components may inhibit the growth of *Lactobacillus* [30]. Therefore, in this study, we not only adopted a fed-batch strategy but also regulated the pH in the early stage of fermentation. In another study, *Lactobacillus acidophilus* IFFI 6005 KLDS 1.0738 also achieved high-density culture in controlled pH batch fermentations [31], which is consistent with our findings. Considering the special value of organic acids [32], the pH was not regulated in the later stage of fermentation and so the pH was reduced to 3.5, and more organic acids were produced, which may be more conducive to application.

Second, previous reports have shown that heat-killed *Lactiplantibacillus plantarum* L-137 [11], *Enterococcus faecalis* [33], and *Lacticaseibacillus rhamnosus* (LR) [12] all improve growth performance in weaned piglets, which is consistent with our findings. This is because these live or heat-killed *Lactobacillus* strains provide a functional component that facilitate nutrient absorption and immunity. Changes in the nutrient composition of the diet were unlikely to result in a significant increase in growth performance of the HKLA group. This is because the heat-killed *Lactobacillus acidophilus* addition was only 600 g/t = 0.6 g/kg, and the ADFI was 1.14 kg. Even if heat-killed *Lactobacillus acidophilus* has an energy content of fat (36 kJ/g) [34], this is not likely to explain the better growth. However, the ADFI of the HKLA group was higher than that of the NC group, and it may be that heat-killed *Lactobacillus acidophilus* can increase the palatability of the feed. The LA group did not achieve the desired feeding effect, and there was no significant difference in growth performance compared with the NC group. However, previous studies have shown that *Lactobacillus acidophilus* can promote animal growth and improve feed conversion [35], which is inconsistent with our findings. This may be related to the high pelleting temperature of the feed, and most of the live bacteria were killed [36]. However, this heat-killed was different from the HKLA group, because the LA group only contained heat-killed bacteria, while the HKLA group contained heat-killed bacteria and metabolites produced during the culture process [37]. In addition, the temperature of heat-killed *Lactobacillus* may also be critical, and the temperature of heat-killed *Lactobacillus* in the HKLA group was 100 °C that higher than the pelleting temperature of the feed, which may have led to the cleavage of the thallus and the release of more beneficial substances.

Third, diarrhea poses a significant challenge in the breeding of weaning piglets [20]. As a probiotic, *Lactobacillus acidophilus* IFFI 6005 plays a crucial role in metabolism, whereby it produces antibacterial substances such as L-lactic acid and acidolin [37], thus inhibiting intestinal bacteria, which can be seen in previous experimental results. Our results suggest that the addition of heat-killed *Lactobacillus acidophilus* IFFI 6005 had the effect of reducing the diarrhea rate in piglets. Another study showed that different doses of heat-killed *Lactobacillus* could reduce the concentrations of TNF-α, TGF-β, and cortisol in serum; improve the immunity of piglets; and reduce the diarrhea rate of piglets, which is also consistent with the results of this trial [12]. This may be related to the ability of heat-killed *Lactobacillus* to regulate the balance of intestinal microbiota and reduce intestinal damage in piglets.

Fourth, it has been demonstrated by researchers that maintaining a well-balanced microbial community structure can effectively prevent the occurrence of intestinal diseases, enhance overall performance, and reduce diarrhea-related indicators [38]. The diversity of gut microbiota is closely associated with host health and metabolic capacity. A decreased diversity of the intestinal microbiota is often considered an indication of intestinal dysbiosis, which can lead to various conditions such as autoimmune diseases, inflammatory bowel diseases, diarrhea, and metabolic disorders. On the other hand, an increased bacterial richness and diversity serve as markers for the establishment of a healthy intestinal microbiota [39]. However, it has also been reported that adding heat-killed *Ligilactobacillus salivarius* strain 189 (HK LS 189) to the diet does not significantly increase the diversity and abundance of gut microbes [13]. Thus, the effects of probiotics on the alpha diversity of the gut microbiota appear to vary depending on the microorganism strain, diet, and environmental factors [40]. There are reports that methane produced by the gastrointestinal tract of ruminant livestock is an important loss in the body’s energy utilization process, usually accounting for more than 10% of the total feed energy, so the reduction of *Methanosphaera* abundance, and possibly the reduction of energy loss, contribute to the improvement of the feed conversion rate [41]. Compared with the NC group, the relative abundance of *Bacillus* was higher in the HKLA group; *Bacillus* can not only produce digestive enzymes, such as amylase and protease, which can stimulate the natural digestion of the host animal and improve growth performance, but also produce bacteriocins to inhibit pathogenic bacteria, which is beneficial to intestinal health [4]. An increase in the abundance of *RB41* in the gut is also advantageous because *RB41* belongs to the genus *Acetobacter*, which is a beneficial bacterium in the intestine that can use carbon sources to produce organic acids to prevent constipation and the production of intestinal gas [42]. *Nitrospira* is capable of metabolizing nitrogen and aromatic compounds and may be involved in the utilization of amino acids and the degradation of toxic substances [43]. Both *Nitrospira* and *MND1* are capable of metabolizing nitrogen and aromatic compounds and may be involved in the utilization of amino acids and the degradation of toxic substances [42,43]. *Collinsella* is an intestinal condition pathogen, and previous studies have shown that it may affect the level of inflammation in the host, so a decrease in its abundance may indicate a decrease in intestinal damage [44]. The addition of heat-killed *Lactobacillus acidophilus* IFFI 6005 to the diet did not affect the content of *Lactobacillus*, and the presence of *Lactobacillus* has the potential to exert inhibitory effects on the growth of intestinal pathogenic bacteria, thereby providing relief from metabolic diseases and intestinal inflammation [45,46]. Studies have shown that heat-killed *Lactiplantibacillus plantarum* b240 [47] and mixed *Lactobacillus* [48] can inhibit the adhesion and invasion of pathogenic bacteria. We speculate that the antibacterial mechanism of heat-killed *Lactobacillus acidophilus* IFFI 6005 is not the same as that of antibiotics, which can selectively inhibit harmful intestinal bacteria without affecting the content of *Lactobacillus*, and that can not only kill pathogenic bacteria through organic acids and bacteriocins, but more importantly, it adheres to intestinal epithelial cells and inhibits the adhesion and invasion of pathogenic bacteria in the intestine by competing with pathogenic bacteria for adhesion sites and nutrients, and alleviates intestinal chaos.

Finally, previous studies have shown that piglets with intestinal inflammation of the small intestine had an increased presence of metabolites in feces due to malabsorption [49]. The results of this study showed that the 10 major differential metabolites were significantly reduced except for a significant increase in guanidine compared to the control group. The piglets in the test group had better digestion and absorption and healthier intestines. Agmatine and (S)-2-hydroxyglutarate are involved in amino acid metabolism, mainly involving lysine, arginine, and proline. Lysine is considered to be the first limiting amino acid in the conventional diet of pigs, and the deficiency or excess of lysine will affect the accumulation of nitrogen and protein metabolism, which, in turn, affects the growth performance and the digestibility of nutrients for pigs [50]. Arginine deficiency has been reported to be one of the key factors limiting the optimal growth performance of piglets [51]. Proline and its derivatives play a unique role in protecting cell structure and function and are considered to be conditioned essential amino acids in young mammals, playing an important role in regulating the inflammatory response and reducing oxidative stress [52,53]. Dl-a-hydroxybutyric acid and 2-deoxy-d-ribose are involved in carbohydrate metabolism, phosphoric acid is involved in energy metabolism, and starch-based polysaccharides in the diet are the main energy source of animals, which plays an important role in the growth and development of animal bodies and directly affects the feed conversion rate [54]. We hypothesize that the differences in these five metabolites affect the digestion and absorption rates of nutrients such as protein and starch in the diet, resulting in different feed conversion rates between the two groups. Chenodeoxycholate and N-(octadecanoyl)sphing-4-enine-1-phosphocholine are involved in lipid metabolism, including primary bile acid biosynthesis and sphingolipid metabolism. Both primary bile acids and sphingomyelins are closely related to cholesterol in the liver, and cholesterol metabolism is related to the antioxidant capacity of mammals and the oxidative stress of the body [39,55,56]. Thiamine monophosphate and thiamine participate in essential vitamin thiamine metabolism, and previous experiments have shown that thiamine levels are upregulated in the test group with higher rates of diarrhea [57]. 7-Methyluric acid is involved in caffeine metabolism, and as a plant alkaloid with detoxification and astringent effects, it can affect diarrhea [35]. We hypothesize that the differences in these five metabolites caused differences in the diarrhea rates between the two groups by affecting the intestinal stress and health of the piglets.

There is increasing evidence of an interconnection between the microbiota and metabolites, which is a key factor in regulating animal metabolism, growth, and development [58,59]. In this study, Spearman’s correlation revealed an association between the abundance of specific bacterial genera and metabolites that were significantly influenced by heat-killed *Lactobacillus acidophilus* IFFI 6005. We hypothesize that the mechanism of the effect of heat-killed *Lactobacillus acidophilus* IFFI 6005 on weaned piglets is that the addition of HKLA improves the intestinal microbial diversity and abundance and reduces the abundance of pathogenic bacteria of piglets. The key differential microorganisms have six genera that can cause differences in ten key metabolites in the intestine.

## 5. Conclusions

In summary, heat-killed *Lactobacillus acidophilus* with broad-spectrum antibacterial activity was fermented and prepared. The findings of this study demonstrated the beneficial effects of adding heat-killed *Lactobacillus acidophilus* on the growth performance, intestinal bacteria, and metabolites of piglets. Furthermore, the potential application of heat-killed *Lactobacillus acidophilus* as a substitute for antibiotics in weaned piglets shows promising prospects. In the future, the effect of heat-killed *Lactobacillus acidophilus* in other animals, the optimal dosage of additives, and more in-depth mechanism exploration should be studied, which will provide valuable insights for the future utilization of heat-killed *Lactobacillus acidophilus* in replacing antibiotics in animal husbandry rearing practices.

## Figures and Tables

**Table 1:** Effect of each treatment group on the performance of weaned piglets (*n* = 24 in each treatment).

Items	NC	PC	LA	HKLA	SEM	*p* Value
BW1 (kg)	14.28	14.36	14.63	14.25	0.37	0.843
BW30 (kg)	35.75 ^a^	37.55 ^b^	36.25 ^a^	38.74 ^b^	0.69	0.045
ADG (g)	715.67 ^a^	773.00 ^bc^	720.67 ^a^	816.33 ^c^	13.36	0.025
ADFI (g)	1090.76 ^a^	1051.28 ^a^	1095.42 ^a^	1142.86 ^b^	18.64	0.0659
F:G	1.52 ^a^	1.36 ^b^	1.52 ^a^	1.40 ^b^	0.02	0.017
Diarrhoea rate (%)	5.83 ^a^	3.33 ^b^	5.14 ^a^	2.64 ^b^	0.15	0.021

Abbreviations: NC = negative control (basal diet); PC = positive control (basal diet + chlortetracycline 380 ppm); LA = live bacteria group (basal diet + *Lactobacillus acidophilus* IFFI 6005 600 g); HKLA = test group (basal diet + heat-killed *Lactobacillus acidophilus* IFFI 6005 600 g); BW1 = BW at 40 days of age; BW30 = BW at 70 days of age; ADG = the average daily weight gain; ADFI = the average daily feed intake; F:G = the ratio of feed to gain; a,b,c = within a row, values with different letter superscripts indicate significant differences (*p* < 0.05).

## Data Availability

The raw sequence data reported in this paper have been deposited in the Genome Sequence Archive in National Genomics Data Center, China National Center for Bioinformation / Beijing Institute of Genomics, Chinese Academy of Sciences (GSA: CRA011798 and OMIX004513) that are publicly accessible at https://ngdc.cncb.ac.cn/gsa and https://ngdc.cncb.ac.cn/omix: accession no.OMIX004513, accessed on 27 August 2024.

## Supplementary Materials

The following supporting information can be downloaded at: https://www.mdpi.com/article/10.3390/ani14172528/s1, Table S1: Ingredient composition and nutrient levels of the basal diet for weaned piglets in this experiment; Table S2: The unique OTUs (Tag ≥ 10) based on Venn analysis of the test; Table S3: The main differential metabolites between the two groups; Table S4: The strains used in this study.; Figure S1: The OTUs differed significantly between the two comparison groups; Materials and methods M1. DNA extraction and PCR amplification.
