# Supplementary material for: Heat-Killed Lactobacillus acidophilus Promotes Growth by Modulating the Gut Microbiota Composition and Fecal Metabolites of Piglets
Source: Animals (Basel). 2024 Aug 30;14(17):2528. doi: 10.3390/ani14172528 (PMC11394466; doi:10.3390/ani14172528)
Supplement: Supplementary file 1 [file animals-14-02528-s001.zip › animals-3135091-supplementary.pdf]

Supplementary materials

Animals

# Heat-Killed *Lactobacillus acidophilus* Promotes Growth by Modulating the Gut Microbiota Composition and Fecal Metabolites of Piglets

Huabiao Miao [1,2,3], Jing Liang [4], Ganqiu Lan [4], Qian Wu [1,2,3], Zunxi Huang [1,2,3*]

1.School of Life Science, Yunnan Normal University, Kunming 650500, China

2.Engineering Research Center for Efficient Utilization of Characteristic Biological Resources in Yunnan, Ministry of Education, Kunming 650500, China

3.Key Laboratory of Yunnan for Biomass Energy and Biotechnology of Environment, Kunming 650500, China

4.Laboratory of Animal Genetics and Breeding, College of Animal Science and Technology, Guangxi University, Nanning 530004, China

* Corresponding authors: Z. Huang. Email: huangzunxi@163.com or huangzunxi@ynnu.edu.cn.

Tel: +86-0871-5920830; Fax: +86 0871-5920952.

**Supplementary figure**

**Supplementary figure S1. The OTUs differed significantly between the two comparison groups.** OTUs = operational taxonomic units; NC = control group; HKLA = test group. The analysis of differences between the two groups used the T-test.

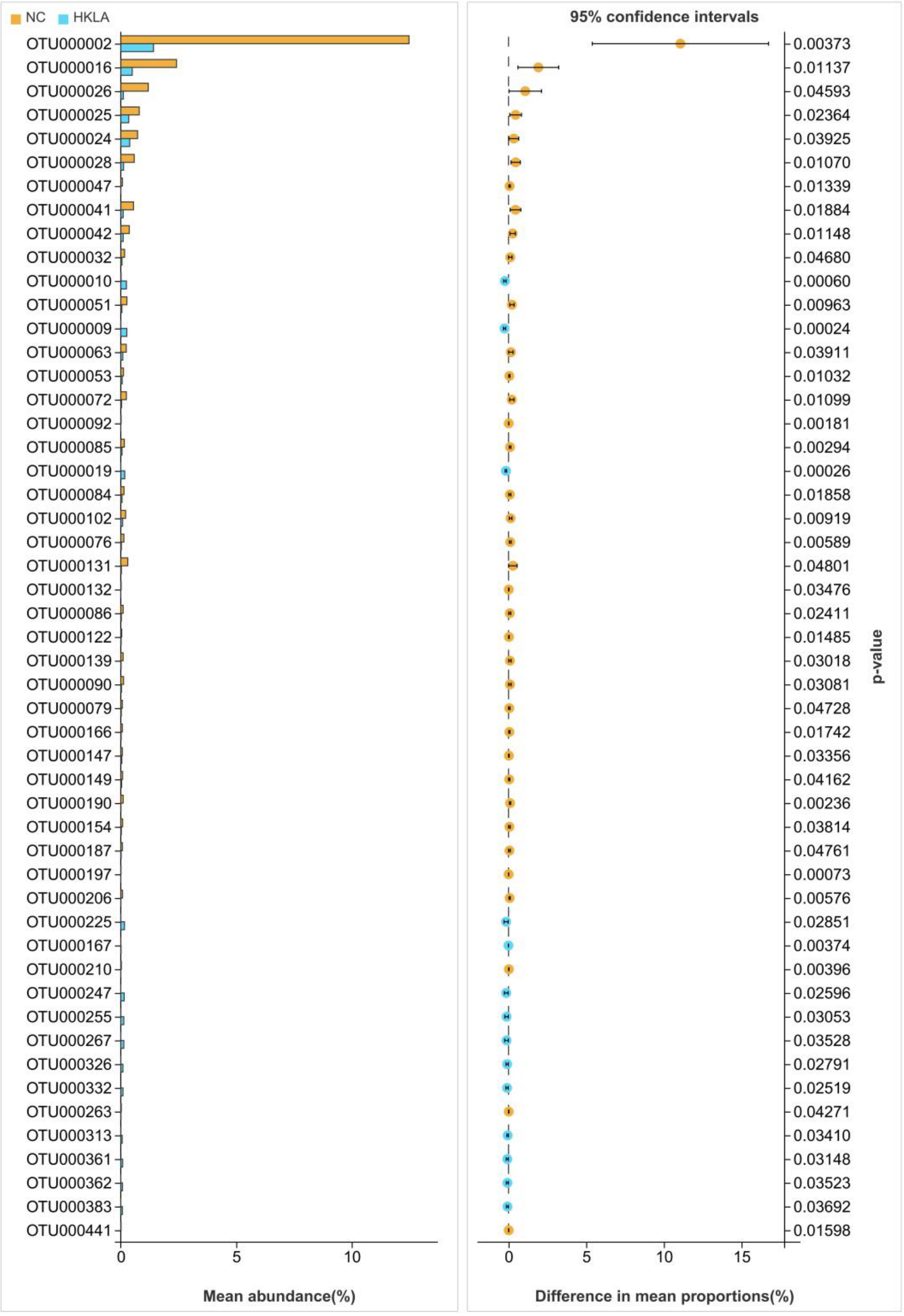

## Supplementary tables

**Supplementary Table S1. Ingredient composition and nutrient levels of the basal diet for weaned piglets in this experiment (%, as-fed basis).**

| Ingredients | Content | Nutrient level[1] | Content |
|---|---|---|---|
| Maize | 60.13 | Digestible energy, MJ/kg | 14.57 |
| Soybean meal (46%) | 18.30 | Dry matter | 88.00 |
| Extruded soybean | 12.00 | Crude protein | 20.12 |
| Fish meal (66%) | 3.00 | Crude fiber | 2.35 |
| Soybean oil | 1.20 | Ash | 1.81 |
| Glucose | 1.20 | Calcium | 0.84 |
| Dicalcium phosphate | 1.00 | Total phosphorus | 0.61 |
| Limestone | 1.00 | Standard total intestinal digestibility of phosphorus | 0.41 |
| *L*-lysine | 0.33 | Standard ileal digestible lysine | 1.12 |
| *DL*-methionine | 0.03 | Standard ileal digestible methionine | 0.31 |
| *L*-threonine | 0.07 | Standard ileal digestible threonine | 0.66 |
| Choline chloride | 0.04 | Standard ileal digestible tryptophan | 0.20 |
| Salt | 0.60 | Standard ileal digestible valine | 0.74 |
| Antioxidants 60 | 0.05 | | |
| Vitamin C | 0.04 | | |
| Acidulant[2] | 0.50 | | |

| | |
|---|---|
| Vitamin premix[3] | 0.08 |
| Mineral premix[4] | 0.20 |
| Compound enzyme[5] | 0.03 |
| Mildew-proof agent[6] | 0.10 |
| Feed attractant[7] | 0.10 |
| Total | 100.00 |

[1] Nutrients levels were calculated values.

[2] Main ingredients are 2-hydroxy-4-methylthiobutanoic acid.

[3] The vitamin premix provided the following per kilogram of diet: vitamin A, 9 000 IU; vitamin D3, 3 000 IU; vitamin E, 20 IU; vitamin K3, 3.0 mg; vitamin B1, 1.5 mg; vitamin B2, 4.0 mg; vitamin B6, 3.0 mg; vitamin B12, 0.2 mg; niacin, 30 mg; pantothenic, 15 mg; folic acid, 0.75 mg; biotin, 0.1 mg.

[4] The mineral premix provided the following per kilogram of diet: Fe, 100 mg; Cu, 150 mg; Mn, 20 mg; Zn, 100 mg; I, 0.3 mg; Se, 0.3 mg.

[5] The compound enzyme provided the following per kilogram of diet: cellulase, 800 IU; $\alpha$-amylase, 1 000 IU; protease, 4 000 IU; phytase, 1 000 FTU.

[6] Main ingredients were mepartricin.

[7] Main ingredients were monosodium glutamate, ethyl maltol, and flavor enhancer.

**Supplementary Table S2. The unique OTUs (Tag ≥ 10) based on Venn analysis of the test.**

| OTU | Tota | NC-1 | NC-2 | NC-3 | NC-4 | NC-5 | NC-6 | HKLA- | HKLA- | HKLA- | HKLA- | HKLA- | HKLA- |
|---|---|---|---|---|---|---|---|---|---|---|---|---|---|

| | l | | | | | | | 1 | 2 | 3 | 4 | 5 | 6 |
|---|---|---|---|---|---|---|---|---|---|---|---|---|---|
| OTU000106 | 1116 | 537 | 546 | 10 | 4 | 7 | 7 | 1 | 3 | 0 | 0 | 1 | 0 |
| OTU000019 | 451 | 1 | 0 | 1 | 2 | 0 | 1 | 48 | 100 | 51 | 50 | 93 | 104 |
| OTU000244 | 368 | 0 | 0 | 0 | 0 | 0 | 0 | 220 | 74 | 34 | 39 | 0 | 1 |
| OTU000191 | 367 | 14 | 13 | 19 | 18 | 156 | 142 | 1 | 2 | 0 | 0 | 0 | 2 |
| OTU000224 | 361 | 147 | 204 | 2 | 1 | 2 | 1 | 0 | 4 | 0 | 0 | 0 | 0 |
| OTU000247 | 358 | 0 | 1 | 0 | 0 | 1 | 0 | 101 | 110 | 66 | 77 | 1 | 1 |
| OTU000267 | 306 | 0 | 1 | 0 | 0 | 1 | 0 | 74 | 51 | 80 | 97 | 2 | 0 |
| OTU000332 | 228 | 0 | 0 | 0 | 0 | 0 | 0 | 67 | 57 | 51 | 52 | 1 | 0 |
| OTU000333 | 220 | 0 | 0 | 0 | 0 | 0 | 0 | 2 | 1 | 3 | 203 | 7 | 4 |
| OTU000361 | 189 | 0 | 0 | 0 | 0 | 0 | 0 | 60 | 41 | 52 | 36 | 0 | 0 |
| OTU000362 | 181 | 1 | 0 | 0 | 0 | 0 | 2 | 42 | 57 | 53 | 25 | 1 | 0 |
| OTU000374 | 175 | 0 | 0 | 0 | 0 | 0 | 0 | 0 | 1 | 2 | 2 | 169 | 1 |
| OTU000383 | 168 | 0 | 1 | 0 | 0 | 0 | 1 | 32 | 33 | 46 | 54 | 0 | 1 |
| OTU000417 | 138 | 1 | 0 | 1 | 0 | 2 | 0 | 38 | 19 | 26 | 51 | 0 | 0 |
| OTU000324 | 134 | 0 | 0 | 0 | 0 | 0 | 0 | 74 | 0 | 10 | 0 | 5 | 45 |
| OTU000381 | 133 | 74 | 48 | 3 | 7 | 1 | 0 | 0 | 0 | 0 | 0 | 0 | 0 |
| OTU000455 | 119 | 0 | 0 | 0 | 0 | 0 | 0 | 2 | 5 | 1 | 6 | 34 | 71 |
| OTU000411 | 108 | 2 | 1 | 1 | 1 | 0 | 0 | 48 | 48 | 2 | 1 | 0 | 4 |
| OTU000496 | 108 | 0 | 0 | 0 | 0 | 0 | 0 | 86 | 8 | 2 | 4 | 0 | 8 |
| OTU000488 | 107 | 1 | 0 | 0 | 0 | 0 | 0 | 28 | 29 | 27 | 22 | 0 | 0 |
| OTU000312 | 94 | 0 | 3 | 4 | 0 | 57 | 30 | 0 | 0 | 0 | 0 | 0 | 0 |

| | | | | | | | | | | | | |
|---|---|---|---|---|---|---|---|---|---|---|---|---|
| OTU000256 | 91 | 27 | 39 | 15 | 2 | 3 | 1 | 1 | 0 | 0 | 0 | 0 | 3 |
| OTU000547 | 90 | 0 | 1 | 0 | 0 | 0 | 0 | 31 | 25 | 22 | 11 | 0 | 0 |
| OTU000492 | 89 | 2 | 1 | 37 | 46 | 1 | 0 | 0 | 0 | 0 | 2 | 0 | 0 |
| OTU000560 | 89 | 0 | 0 | 1 | 0 | 0 | 0 | 19 | 22 | 24 | 21 | 0 | 2 |
| OTU000562 | 89 | 0 | 1 | 32 | 56 | 0 | 0 | 0 | 0 | 0 | 0 | 0 | 0 |
| OTU000436 | 87 | 47 | 37 | 2 | 1 | 0 | 0 | 0 | 0 | 0 | 0 | 0 | 0 |
| OTU000512 | 85 | 0 | 3 | 2 | 3 | 43 | 33 | 0 | 0 | 0 | 0 | 0 | 1 |
| OTU000585 | 76 | 0 | 1 | 0 | 0 | 1 | 0 | 10 | 21 | 21 | 22 | 0 | 0 |
| OTU000619 | 75 | 1 | 0 | 0 | 0 | 0 | 0 | 19 | 16 | 15 | 24 | 0 | 0 |
| OTU000568 | 73 | 0 | 0 | 0 | 0 | 0 | 0 | 35 | 36 | 0 | 1 | 0 | 1 |
| OTU000627 | 73 | 0 | 0 | 0 | 0 | 0 | 0 | 9 | 19 | 21 | 23 | 1 | 0 |
| OTU000644 | 68 | 0 | 0 | 0 | 0 | 0 | 0 | 1 | 1 | 0 | 2 | 64 | 0 |
| OTU000649 | 68 | 0 | 0 | 0 | 0 | 0 | 0 | 17 | 17 | 18 | 16 | 0 | 0 |
| OTU000513 | 67 | 0 | 0 | 1 | 1 | 0 | 0 | 24 | 41 | 0 | 0 | 0 | 0 |
| OTU000646 | 65 | 0 | 0 | 0 | 0 | 0 | 0 | 23 | 19 | 19 | 4 | 0 | 0 |
| OTU000629 | 63 | 0 | 0 | 0 | 0 | 1 | 1 | 28 | 30 | 0 | 1 | 0 | 2 |
| OTU000664 | 63 | 0 | 0 | 0 | 0 | 0 | 0 | 10 | 20 | 19 | 14 | 0 | 0 |

Abbreviations: OTUs = operational taxonomic units; NC = control group; HKLA = test group.

**Supplementary Table S3. The main differential metabolites between the two groups.**

| Compound_ID | MS2_name | Content changes |
|---|---|---|

| | | NC_vs_HKLA |
|---|---|---|
| M217T332_POS | beta-glycerophosphate | down |
| M373T106_POS | beta-muricholic acid | down |
| M100T402_POS | 2-piperidone | down |
| M269T177_POS | 2'-hydroxy-5'-methyl-2-methoxychalcone | down |
| M146T48_POS | 4-hydroxyquinoline | down |
| M139T310_2_POS | 4-imidazoleacrylic acid | up |
| M486T163_POS | 4-quinazolinamine, 7-[3-(dimethylamino)propoxy]-2-(hexahydro-4-methyl-1h-1,4-diazepin-1-yl)-6-methoxy-n-(1-methyl-4-piperidinyl)- | down |
| M118T402_2_POS | 5-aminovaleric acid | up |
| M183T113_POS | 7-methyluric acid | up |
| M136T173_1_POS | Adenine | down |
| M114T41_POS | Agmatine | up |
| M182T163_POS | Anhydroecgonine methyl ester | down |
| M187T62_POS | Bis-(acetyl)diaminopentane | down |
| M387T146_POS | Bufalin | down |
| M151T175_POS | D-xylose | up |
| M252T149_POS | Deoxyadenosine | down |
| M136T319_POS | Dl-octopamine | down |
| M253T385_POS | His-Pro | down |
| M415T175_POS | Leukotriene b4 3-aminopropylamide | down |

| | | |
|---|---|---|
| M145T321_1_POS | N-acetylcadaverine | down |
| M101T402_1_POS | N-nitrosopyrrolidine | down |
| M521T33_POS | N-palmitoyl-d-sphingosine | up |
| M159T289_2_POS | Nonanoic acid | down |
| M297T29_POS | Oleic acid methyl ester | down |
| M247T163_POS | Oxymatrine | down |
| M413T106_POS | Palmitoyl 3-carbacyclic phosphatidic acid | down |
| M209T62_POS | Pilocarpine | down |
| M146T270_POS | Spermidine | up |
| M265T367_POS | Thiamine | up |
| M122T367_POS | Thiamine monophosphate | up |
| M128T321_POS | Tillam | down |
| M142T289_POS | Tropine | down |
| M446T36_POS | Trp-Ile-Lys | down |
| M182T319_2_POS | Tyrosine | down |
| M187T251_2_POS | Undecanoic acid | down |
| M171T118_NEG | (-)-hydroxycitric acid lactone | down |
| M245T361_2_NEG | (+)-abscisic acid | down |
| M147T414_1_NEG | (S)-2-Hydroxyglutarate | up |
| M155T140_NEG | 1,3-dimethylbarbituric acid | down |
| M188T214_NEG | 1h-indole-3-propanoic acid | down |
| M179T364_NEG | 2-deoxy-d-ribose | up |

| | | |
|---|---|---|
| M117T161_2_NEG | 2-hydroxy-2-methylbutyric acid | down |
| M329T326_1_NEG | 3,2'-dihydroxy-4,4',6'-trimethoxychalcone | down |
| M139T334_2_NEG | 3,5-dihydroxybenzyl alcohol | down |
| M780T100_NEG | 3.alpha.-hydroxy-7-oxo-5.beta.-cholanic acid | up |
| M162T45_NEG | 5-hydroxy-3,4-dihydro-2(1h)-quinolinone | down |
| M186T184_NEG | 7-keto-8-aminopelargonic acid | up |
| M435T56_NEG | Artocaprin | down |
| M784T172_NEG | Chenodeoxycholate | up |
| M389T100_3_NEG | Cholic acid | down |
| M103T206_2_NEG | Dl-a-hydroxybutyric acid | up |
| M297T165_NEG | Enterolactone | down |
| M337T27_NEG | Eplerenone hydroxy acid | down |
| M135T128_NEG | Glabridin | down |
| M448T240_2_NEG | Glycochenodeoxycholate | up |
| M129T414_NEG | L-2-hydroxyglutaric acid | down |
| M128T322_3_NEG | L-pyroglutamic acid | up |
| M375T69_NEG | Lithocholic acid | down |
| M303T414_2_NEG | Melphalan | up |
| M309T27_2_NEG | Mestranol | down |
| M147T149_NEG | Mevalonic acid | up |
| M766T146_NEG | N-(octadecanoyl)sphing-4-enine-1-phosphocholine | up |
| M172T214_NEG | N-acetyl-d-norleucine | down |

| | | |
|---|---|---|
| M180T320_2_NEG | N-acetyl-l-tyrosine | down |
| M504T205_NEG | N-octanoylsphingosine-1-phosphate | down |
| M782T174_NEG | N-stearoyltaurine | down |
| M97T86_NEG | Phosphoric acid | up |
| M231T275_NEG | Thr-Leu | down |
| M287T259_NEG | Trans-dehydroandrosterone | up |
| M215T237_NEG | Val-Val | down |

Abbreviations: $p < 0.05$ and $|\log_2(FC)| > 2$; NC = control group; HKLA = test group.

**Supplementary Table S4. The strains used in this study.**

| Stains | Preservation number | Function | Source |
|---|---|---|---|
| *Bacillus subtilis* | CGMCC 1.12938 | Activity studies | China General Microbiological Culture Collection Center |
| *Bacillus licheniformis* | CGMCC 1.7677 | Activity studies | |
| *Bacillus coagulans* | CGMCC 1.10823 | Activity studies | |
| *Enterococcus faecalis* | CGMCC 1.2135 | Activity studies | |
| *Lactobacillus acidophilus* | CGMCC 1.3342 | Activity studies | |
| *Staphylococcus aureus* | CGMCC 1.184 | Indicator bacteria | |
| *Salmonella enterica* | CGMCC 1.755 | Indicator bacteria | |
| *Escherichia coli* | CICC 10899 | Indicator bacteria | China Center of Industrial Culture |
| *Clostridium perfringens* | CICC 22949 | Indicator bacteria | |

**Supplementary Materials and methods**

**Supplementary Materials and methods M1.** DNA extraction and PCR amplification. Total fecal microbial DNA was extracted using a Fecal Genomic DNA Extraction Kit (Omega Bio-Tek, Norcross, GA, USA) and was then purified and tested for concentration and purity using a NanoDrop 2 000 (Thermo Fisher Scientific, USA). Bacterial 16S rDNA (V3 and V4 regions) was amplified by PCR using total fecal DNA as a template. Bacterial universal primers were used for the upstream and downstream primers: 338F (5`-ACTCCTACGGGAGGCAGCAG-3`) and 806R (5`-GGACTACHVGGGTWTCTAAT-3`). The amplification system was as follows: 12.5 μL of 2×FastPfu mix, 1.0 μL of each primer (5 μM), 10 ng of template DNA, and $ddH_2O$ supplemented to 25.0 μL. The PCR parameters were as follows: 94 °C for 5 min; 30 cycles of 94 °C for 30 s, 53 °C for 45 s, 72 °C for 50 s; 72 °C for 12 min; and 4 °C cool down. The PCR products were assessed by 2% agarose gel electrophoresis, and the resolved bands were cut, recovered, and sequenced by an Illumina MiSeq PE300.
